# Measurements of elemental iodine in soy sauces in Taiwan using a modified microplate method

**DOI:** 10.3389/fendo.2023.1058695

**Published:** 2023-03-09

**Authors:** Chun-Jui Huang, Lin-Hsuan Lee, Cheng-Pin Cheng, Shan-Fan Yao, Harn-Shen Chen, Chii-Min Hwu, Kam-Tsun Tang, Fan-Fen Wang, Chiao-Wei Shih, Chen-Chang Yang, Wen-Sheng Huang

**Affiliations:** ^1^ Division of Endocrinology and Metabolism, Department of Medicine, Taipei Veterans General Hospital (TVGH), Taipei, Taiwan; ^2^ Faculty of Medicine, School of Medicine, National Yang Ming Chiao Tung University (NYCU), Taipei, Taiwan; ^3^ Institute of Public Health, School of Medicine, National Yang Ming Chiao Tung University (NYCU), Taipei, Taiwan; ^4^ Institute of Food Safety and Health Risk Assessment, School of Pharmaceutical Science, National Yang Ming Chiao Tung University (NYCU), Taipei, Taiwan; ^5^ Department of Nuclear Medicine, Taipei Veterans General Hospital, Taipei, Taiwan; ^6^ Department of Medicine, Yangming Branch, Taipei City Hospital, Taipei, Taiwan; ^7^ Institute of Environmental & Occupational Health Sciences, School of Medicine, National Yang Ming Chiao Tung University (NYCU), Taipei, Taiwan; ^8^ Department of Occupational Medicine and Clinical Toxicology, Taipei Veterans General Hospital, Taipei, Taiwan; ^9^ Department of Nuclear Medicine, Cheng-Hsin General Hospital, Taipei, Taiwan

**Keywords:** iodine, low iodine diet, soybean, soy sauce, Taiwan

## Abstract

**Background:**

Soy sauce is widely used in a variety of Asian dishes to enhance flavor. Soybean and most soybean products, including soy sauces, are listed as prohibited foods in a low iodine diet. However, the iodine content in soy sauces is largely unknown. The aim of this study was to determine the iodine content in domestic soy sauces in Taiwan.

**Methods:**

Twenty-five different kinds of soy sauces were diluted with distilled water and with a dilution factor of fifty or above. Iodine concentrations of the diluted samples were measured colourimetrically based on the Sandell-Kolthoff reaction by a modified microplate method. All the measurements were repeated twelve times on three different days for determination of mean and standard deviation (SD), and coefficients of variance (CV). Serial dilution and recovery tests were also performed for validation. The results were confirmed by an inductively coupled plasma mass spectrometry (ICP-MS) method.

**Results:**

Among the twenty-five surveyed soy sauces, most of them (n=22) were iodine-free (<16 ug/L, and thus un-detectable). The iodine concentrations (mean ± SD) of the three iodine-containing soy sauces were 2.7 ± 0.1, 5.1 ± 0.2, and 10.8 ± 0.6 mg/L, respectively. The inter-assay, intra-assay and total CVs were all <5.3% for the modified microplate method. The results obtained by ICP-MS were consistent with those of the modified microplate method. The recovery rates in the serial dilution test and recovery test ranged from 94.7% to 118.6%. Two of the three iodine-containing soy sauces were supplemented with kelp extract, while the other one without kelp extract had the highest amount of salt among the three iodine-containing soy sauces. Therefore, we postulate that iodized salt instead of kelp extract is the source of higher iodine content in that sauce.

**Conclusion:**

The results suggest that most soy sauces are iodine-free and may be allowed during low iodine diets.

## 1 Introduction

A low iodine diet (LID) has been suggested 2 weeks prior to radioiodine administration for patients with differentiated thyroid carcinoma after thyroidectomy to enhance radioiodine uptake in remnants or residual tumor to ensure effective ablation or treatment (1, 2). Available data indicate that the alcohol soluble component in soybeans inhibits thyroid absorption of iodine and thus interferes with radioiodine thyroid uptake, leading to reduced efficacy of radioiodine therapy (3). Soy sauces are made from soybeans and are not recommended during a LID (2, 4–6). Soybeans *per se* do not contain iodine, but soy sauces may contain iodine if iodized salts are added during the fermentation process or if food additives such as seaweeds or yeasts are added during processing to enhance the flavor of soy sauces (3, 7).

To carry out the LID appropriately, it is important to determine the iodine contents in local foods. With the same food type, the iodine content may vary greatly in different regions due to differences in soil conditions, animals’ diet and iodine contents of ingredients in processed foods (8). For instance, the milk iodine content is 343 μg/kg in the United States, 220 μg/kg in Israel and 160 μg/kg in Norway (9–11). Even within the same country, the milk iodine content also varies in different regions (12). A survey of food composition in Japan had determined iodine content in 8 types of soy sauces and found 4 of them containing iodine (10 μg/kg in 3 types and the other one was 7,500 μg/kg), while the other 4 were iodine-free (13). However, in a very detailed food database in the United States, iodine content (10 μg/kg) was detected in only one type of soy sauce (9). There are more than 20 types of soy sauces in Taiwan but none of them has been tested for iodine contents. Some regional databases for iodine contents in foods and dietary supplements were established, but this is not the case in Taiwan (9, 10, 14); this is confusing for patients because they cannot know whether soy sauces, an important condiment in Taiwan, can be consumed during a LID or not.

Measuring iodine contents in foods is more complex than determining urinary iodine concentrations (UIC). In Taiwan, measurements of UIC in iodine nutritional studies have been carried out by a modified microplate method based on the Sandell–Kolthoff (S-K) reaction during the past 10 years (15, 16). While highly accurate in measuring UIC, one of the drawbacks of the S-K method is its limitation in determining iodine content in complex matrixes such as breast milk and foods. Therefore, we recently validated an inductively coupled plasma mass spectrometry (ICP-MS) method using alkali dilution to measure UIC (17–19). Others have previously reported on ICP-MS method for the measurement of iodine in breast milk and foods (20–22). Whether iodine content in soy sauces could be determined by the S-K method or ICP-MS method needs to be clarified.

The aim of the study was to determine the iodine contents of Taiwanese marketing soy sauces and to compare the performance of the S-K reaction and the ICP-MS method.

## 2 Materials and methods

### 2.1 Preparation of soy sauces

A total of 25 soy sauces including some imported and exported brands were collected from supermarkets in Taiwan. The salt content of each brand of soy sauces was converted to the same units according to their labeling. Iodine concentrations were determined colourimetrically by a modified microplate method based on the Sandell-Kolthoff reaction (15, 16). Samples were also measured by an ICP-MS method as previously described (17–19).

### 2.2 Iodine measurement protocol

The protocol of determining iodine content in soy sauces was similar to what had been previously used to measure UIC except for those needed to be diluted by distilled water to allow measurable light transmission of absorbance at the final step. In brief, the digestion step was performed by pipetting 23 μL of standard iodine solutions and sampling into a 96-well reaction plate (Applied Biosystems, Foster City, CA, USA), followed by addition of 46 μL of ammonium persulfate solution (freshly prepared, 1.35 mol/L; Sigma-Aldrich, St. Louis, MO, USA) to each well. The microplate was covered with a 96-well full plate cover, and the contents were digested in the GeneAmp^®^ PCR System 9700 Fast Thermal Cycler (Applied Biosystems) with a program of 95°C for 30 min and 4°C for 5 min. After digestion, the microplate was centrifuged at 1000 rpm for 3 min. The S-K reaction step was then performed. From the resulting digests, 50 µL of aliquots were transferred to the corresponding wells of a 96-well reading plate (MicroWell^®^; Nalge Nunc International, Rochester, NY, USA), in which 100 μL of arsenious acid solution (0.05 mol/L; Sigma-Aldrich) had been preloaded. After mixing the solution by shaking the plate in the microplate reader (Tecan Infinite^®^ F50; Tecan Group Ltd., Männedorf, Switzerland), 50 μL of ceric ammonium sulfate solution (0.019 mol/L; Wako Pure Chemical Industries Ltd, Osaka, Japan) was added into each well using a multichannel pipette as quickly as possible. The absorbance of the reaction mixture was read at 405 nm after incubating at room temperature (~25°C) for 30 min. The calibration curve was plotted for each plate by plotting the optical density values versus the concentration of standards. Sample concentrations were interpolated from the calibration curve (range: 0-400 ug/L). The detection limit is 1.6 ug/L. Quality control samples provided by the Ensuring the Quality of Urinary Iodine Procedures (EQUIP) program were tested in each run to verify the accuracy and the success of the digestion.

### 2.3 Validation of the iodine measurements

The tested samples were duplicated 12 times on 3 different days to determine the mean and standard deviation (SD), intra-assay, inter-assay and total coefficients of variance (CV). To validate the microplate method in determining iodine content in soy sauces, further experiments including the serial dilution and recovery tests were performed. The previously 100-fold or 50-fold diluted samples were further serially diluted (1, 0.8, 0.6, 0.4, and 0.2) to determine the recovery rate and linearity. The recovery test was performed twice on 2 different days and the soy sauces were measured twice to evaluate the iodine concentration before adding additional iodine. The potassium iodate solution was prepared by mixing 0.843 g of potassium iodate with water and diluting to solutions with iodine concentrations of 5 μg/L, 10 μg/L, and 15 μg/L (**Table S1**). One volume of each iodate solution (5 μg/L, 10 μg/L, or 15 μg/L) was added to 9 volumes of sample after being appropriately diluted 100-fold or 50-fold.

### 2.4 Statistical analysis

Statistical analysis was performed using Statistical Package for the Social Sciences (SPSS) software, version 24.0 (IBM Corp., Armonk, NY, USA). Mann-Whitney U test was performed for comparison of salt content between the iodine-containing and iodine-free soy sauces. *P* <0.05 was served as statistically significant.

## 3 Results

**Figure 1:**
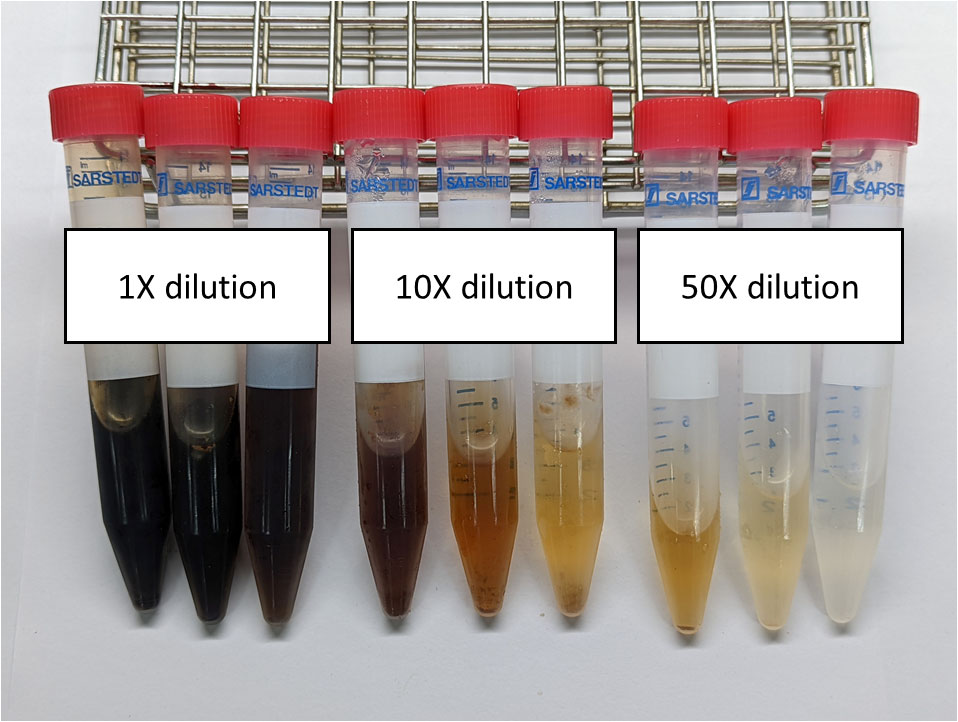
The appearance of the three iodine-containing soy sauces when diluted with different dilution factors.

Samples with different dilution factors were measured by the S-K method to determine the most appropriate dilution factor to yield consistent results. While the samples were diluted with a dilution factor of 10 or less, some of the samples still appeared blackish and the absorbance was severely disturbed by the pigmentation. As diluted 50 times above the original samples, the light could be transmitted through the samples and the absorbance could be obtained accurately as shown in **Figure 1**.

**Figure 2:**
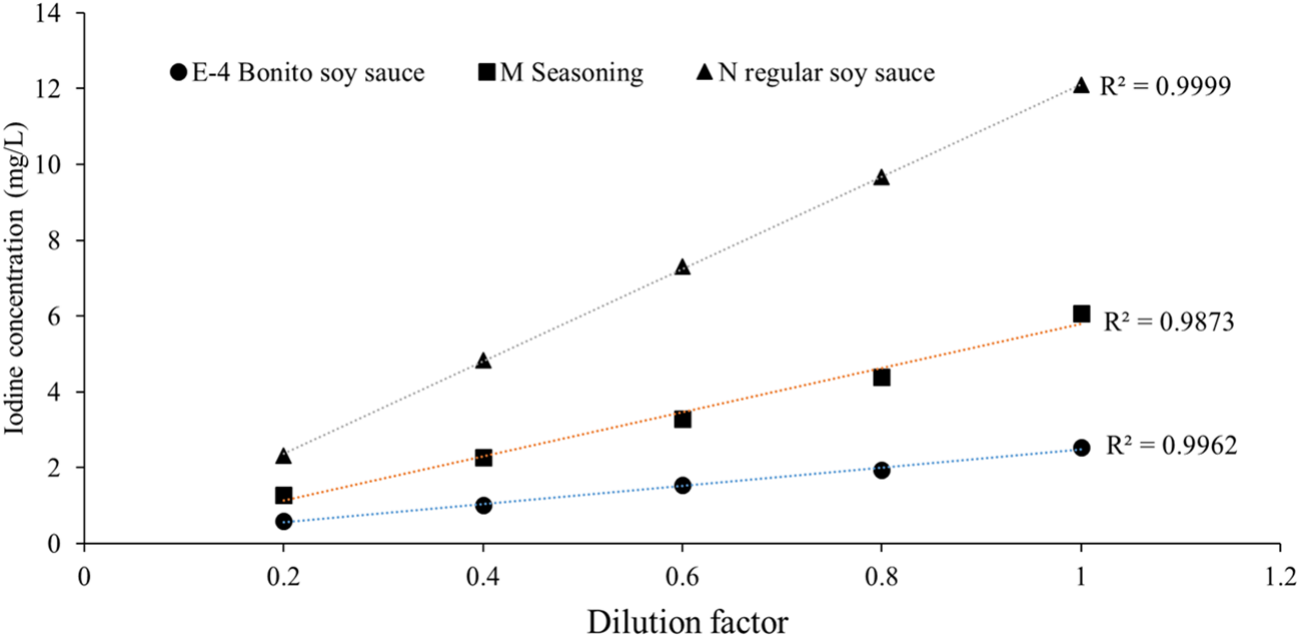
The linearity of iodine concentrations by serial dilution of three soy sauces.

The iodine contents in 25 different domestic soy sauces are summarized in **Table 1**. Only 3 of them contained iodine, while the other 22 were iodine-free. The lowest detectable iodine concentration in the soy sauces was 16 μg/L (**Table S1**). Of the 3 iodine-containing soy sauces, iodine concentrations were determined by a dilution factor of 100 or 50 and the results were 2.7 ± 0.1, 5.1 ± 0.2, and 10.8 ± 0.6 mg/L, respectively (**Tables 2**; **S1**). The inter-assay, intra-assay and total CVs were all <5.3%. The CVs using S-K and ICP-MS methods were 4.7%, 2.9%, and 2.7%, respectively. The mean recovery rate of the 3 iodine-containing soy sauces at serial dilution were 103.4%, 95.1%, and 99.1%, respectively (**Table 3**). The coefficients of linearity were >0.999, 0.987, and 0.996, respectively (**Figure 2**). The iodine recovery rates of the 3 iodate-added samples were 118.6%, 94.7% and 99.4%, respectively (**Table 4**).

**Table 1:** Iodine-containing soy sauces and iodine-free soy sauces.

Iodine-containing or iodine-free	Brand	Item
Iodine-free soy sauces	A	A-1 Regular soy sauce A-2 Regular soy sauce

	B	B-1 Regular soy sauce B-2 Regular soy sauce B-3 Thick soy sauce B-4 Thick soy sauce
	C	C-1 Vegetarian oyster oiled soy sauce C-2 Regular soy sauce C-3 Undercoater soy sauce
	D	D-1 Regular soy sauce D-2 Thick soy sauce
	F	F-1 Regular soy sauce F-2 Regular soy sauce
	G	G-1 Black bean sauce (Thick) G-2 Black bean sauce (Light) G-3 Black bean sauce
	H	H-1 Black bean sauce (Pure) H-2 Black bean sauce (Thick)
	I	I Oyster sauce
	J	J Vegetarian Oyster Sauce
	K	K Regular soy sauce
	L	L Regular soy sauce
Iodine-containing soy sauces	E	E Bonito soy sauce
	M	M Seasoning soy sauce
	N	N Regular soy sauce

**Table 2:** Iodine concentration of the three iodine-containing soy sauces.

Samples		E	M	N
Dilution factor		100	50	50
Measurements (mg/L)	1^st^	2.5	4.8	10.3
	2^nd^	2.8	4.7	11.4
	3^rd^	2.7	5.4	10.0
	4^th^	2.8	5.2	11.2
	5^th^	2.8	5.3	12.1
	6^th^	2.8	5.4	10.9
	7^th^	2.8	4.9	10.5
	8^th^	2.6	4.9	10.5
	9^th^	2.7	5.0	10.6
	10^th^	2.4	5.0	10.6
	11^th^	2.6	5.0	10.6
	12^th^	2.6	5.0	10.6
Mean (mg/L)		2.7	5.1	10.8
SD (mg/L)		0.14	0.23	0.57
Inter-CV (%)		0.78	4.1	4.7
Intra-CV (%)		5.3	3.0	4.8
Total CV (%)		5.0	4.5	5.3

E. Bonito soy sauce; M, Seasoning soy sauce; N, Regular soy sauce.

SD, Standard deviation; CV, coefficients of variance.

**Table 3:** Serial dilution of soy sauce samples within water.

Sample	Dilution factor	Iodine concentration (mg/L)		
		Measured	Expected	Recovery %
E	1	2.5		
	0.8	1.9	2.0	95.5
	0.6	1.5	1.5	101.8
	0.4	1.0	1.0	100.4
	0.2	0.6	0.5	116.0
M	1	6.1		
	0.8	4.4	4.9	90.6
	0.6	3.3	3.6	90.6
	0.4	2.3	2.4	93.8
	0.2	1.3	1.2	105.5
N	1	12.1		
	0.8	9.7	9.7	100.0
	0.6	7.3	7.3	100.7
	0.4	4.8	4.8	99.8
	0.2	2.3	2.4	96.1

E, Bonito soy sauce; M, Seasoning soy sauce; N, Regular soy sauce.

**Table 4:** Recovery of iodine added to soy sauces.

	Iodine concentration (mg/L)			
Sample	Source	Expected	Measured	Recovery (%)
E	2.53	3.8	4.5	118.6
M	7.0	8.5	8.1	94.7
N	12.1	13.5	13.46	99.4

E, Bonito soy sauce; M, Seasoning soy sauce; N, Regular soy sauce.

Among the 3 iodine-containing soy sauces, 2 of them contained kelp extract. The salt (NaCl) contents of the 3 iodine-containing soy sauces were 5120, 9255, and 1340 mg per 100 mL, while the average salt content of the other iodine-free soy sauces was 4252 ± 1217.7 mg per 100 mL (**Tables 5**; **S1**).

**Table 5:** Labeling of the three iodine-containing soy sauces.

Ingredients/sample	E	M	N
Additives with high iodine content	Kelp extract	–	Kelp extract
Calories (kcal/100 mL)	143	46	42
Protein (g/100 mL)	6.9	7.1	1.9
Fat (g/100 mL)	0	0	0
Carbohydrate (g/100 mL)	28.9	4.4	8.6
Sodium (mg/100 mL)	5120	9255	1340

E, Bonito soy sauce; M, Seasoning soy sauce; N, Regular soy sauce. - means there is not any.

## 4 Discussion

The iodine content in soy sauces has not been reported thoroughly in the literature. The current observation, to our knowledge, appears to be the first study to comprehensively clarify the iodine contents in several soy sauces marketed in Taiwan. Information regarding iodine content in foods is very limited in Taiwan. As of yet, the iodine content has only been reported for the herbal cuisine soup (median iodine concentration 23.1 μg/L), post-partum teas (<10 μg/L), dairy products and milk-alternative drinks (mean iodine concentration of whole milk, low-fat milk, flavored milk and milk drinks, milk alternative drinks:210.4 μg/L, 263.2 μg/L, 100.0 μg/L, 65.6 μg/L, and <1 μg/L, respectively) (23, 24) (**Table S1**). This study provides additional data on iodine nutrition of local foods in Taiwan, which provides useful information for patients scheduled for a LID diet.

The current study shows that ingestion of 4~20 mL of the 3 iodine-containing soy sauces (iodine content 2.7, 5.1, and 10.8 mg/L, respectively) could exceed the LID recommended criteria, *i.e.*, an iodine intake <50 µg/day (1, 2). It is thus reasonable to recommend against their use during the preparation of LID. Notably, LID does not mean “no” iodine diet. Patient’s convenience, diet habit and well-being are also of major importance in addition to therapeutic efficacy when setting up dietary guidelines. From the clinical point of view, iodine-free soy sauces can be permitted for LID preparation. First, radioiodine uptake would not be impaired by pre-administration of such soybean products per se (25). Second, soy sauces used as seasonings in Asian food are not ingested in large amounts. Lastly, the flavor of soy sauces is so popular in the general population that their use cannot be readily avoided. Therefore, we recommend to allow the consumption of iodine-free soy sauces identified in the current study during the 2 weeks’ LID period.

The source of iodine content in iodine-containing soy sauces might come from food additives, as found in the 2 brands of soy sauces that contain kelp extracts. Kelp is rich in elemental iodine and is often used to provide additional umami in soy sauces (13). Based on the findings presented here, most soy sauces do not contain iodine and can be consumed during a LID. The other iodine-containing soy sauce that did not contain kelp extract had a much higher salt content as compared to iodine-free soy sauces (9,255 mg/100 mL *vs.* 4,252 mg/100 mL). Therefore, the source of elemental iodine might have originated from the added iodized salt. According to the Taiwan Food and Drug Administration, the iodine in salt fortified with potassium iodide or potassium iodate was 12–20 mg/kg before June, 2017 (26). However, around two thirds of the salts available in the domestic markets are non-iodized based on a salt survey in 2012 (27). This might be the reason that most of the salts added in soy sauces are iodine-free. While we do not have information on the manufacturing processes of the soy sauces, we assume that iodized salt rather than kelp extract has been used during processing and was thus responsible for the high iodine-content in the soy sauce. We strongly recommend that soy sauce manufacturers should be required to indicate the iodine content on the product label.

Measurements of iodine content in soy sauces poses certain difficulties compared to UIC measurements because soy sauces have a blackish color due to the fermentation processes of soy beans or the additives such as caramel color. When absorbance was measured at the final step of the S-K reaction, light needs to be transmittable and thus a high dilution factor is compulsory. Typically, when UIC is measured by the S-K method, urine samples do not need to be diluted unless the concentration is outside the range of the calibration curve. Even if dilution is needed, the dilution factor of UIC measurements is usually below 10. In this study, we found that a dilution factor of 50 or above enabled us to achieve a highly accurate result using the S-K method. To our knowledge, this is the first time that iodine content of soy sauces was determined by the S-K reaction. Being prudent, we performed tests including the precision test, serial dilution test, and recovery tests and then obtained the CVs, recovery rates and linearity to validate the results. In addition, we also measured iodine content in soy sauces using ICP-MS and the obtained results were highly consistent with those of the S-K method. Notably, the pipeline and cone orifice of ICP-MS would clog easily after measurements, probably due to the metal ions and volatile compounds in the soy sauces. The ICP-MS method used in the present study employed triton X-100 and 0.5% ammonia solution to prepare the samples into aqueous solution with a final volume 100 times of the original sample (17–19). Tellurium (^128^Te) was the internal standard. Microwave digestion was not needed when UIC was measured by ICP-MS but this needed to be modified when iodine contents were measured in complex matrixes such as soy sauces (22, 28). Furthermore, microwave digestion is time-consuming and is not routinely applicable in ICP-MS measurements. The ammonium persulfate used in the S-K method is able to digest the interfering substances in soy sauces. Based on these considerations and the results presented here, the S-K method can be used to measure the iodine content in soy sauces after appropriate dilution.

There are some limitations in this study. First, not all soy sauces marketed in Taiwan were tested in this study. Second, neither the manufacturing process of soy sauces nor the types of salt (iodized or non-iodized) added during fermentationis known. We postulate that kelp extract and iodized salt at least in part contribute to the iodine content in iodine-containing soy sauces. We suggest that the S-K method can be used to measure the iodine content in soy sauces after appropriate dilution. Finally, further modifications of the analytical procedures may permit ICP-MS to be used to measure the iodine content in complex matrixes such as soy sauces in the future.

In conclusion, except for certain special additives such as kelp extracts or iodized salts, most domestic soy sauces in Taiwan are iodine-free.

## Data availability statement

The raw data supporting the conclusions of this article will be made available by the authors, without undue reservation.

## Author contributions

C-JH and W-SH designed the study. L-HL, C-PC, C-WS performed the iodine measurements. L-HL, C-PC, S-FW, H-SC, C-MH, K-TT, F-FW, C-WS, C-CY analyzed and interpreted the data. C-JH, L-HL, C-PC prepared the manuscript. C-CY and W-SH critically revised the manuscript. All authors contributed to the article and approved the submitted version.
