# Supplementary material for: Measurements of elemental iodine in soy sauces in Taiwan using a modified microplate method
Source: Front Endocrinol (Lausanne). 2023 Mar 9;14:1058695. doi: 10.3389/fendo.2023.1058695 (PMC10054453; doi:10.3389/fendo.2023.1058695)
Supplement: Supplementary file 1 [file Table_1.pdf]

Supplementary Table 1. Comparative table for conversion of units.

Section	Description	Original value and unit	Conversion to μg/L
2.3 Validation of the iodine measurements	iodate solution	5 μg/L, 10 μg/L, or 15 μg/L	5 μg/L, 10 μg/L, or 15 μg/L
3 Results (2nd paragraph)	lowest detectable iodine concentration in the soy sauces	16 μg/L	16 μg/L
3 Results (2nd paragraph)	iodine concentrations results of the 3 iodine containing soy sauce	2.7 ± 0.1, 5.1 ± 0.2, and 10.8 ± 0.6 mg/L	2700 ± 100, 5100 ± 200, and 10800 ± 600 μg/L
3 Results (3rd paragraph)	the salt (NaCl) contents of the 3 iodine-containing soy sauces	5120, 9255, and 1340 mg per 100 mL	51200000, 92550000, and 13400000 μg/L
3 Results (3rd paragraph)	The averaged salt content of the other iodine-free soy sauces	4252 ± 1217.7 mg per 100 mL	42520000 ± 12177000 μg/L
4 Discussion (1st paragraph)	herbal cuisine soup and post-partum teas	23.1 μg/L <10 μg/L	23.1 μg/L <10 μg/L
4 Discussion (1st paragraph)	whole milk, low-fat milk, flavored milk and milk drinks, milk alternative drinks	210.4 μg/L, 263.2 μg/L, 100.0 μg/L, 65.6 μg/L, and <1 μg/L	210.4 μg/L, 263.2 μg/L, 100.0 μg/L, 65.6 μg/L, and <1 μg/L
